# Reflections on the opportunities and challenges of applying experience‐based co‐design (EBCD) to phase 1 clinical trials in oncology

**DOI:** 10.1111/hex.14068

**Published:** 2024-06-27

**Authors:** Nils Graber, Nina Canova, Denise Bryant‐Lukosius, Glenn Robert, Blanca Navarro‐Rodrigo, Lionel Trueb, George Coukos, Manuela Eicher, Tourane Corbière, Sara Colomer‐Lahiguera

**Affiliations:** ^1^ Institute of Higher Education and Research in Healthcare (IUFRS), Faculty of Biology and Medicine University of Lausanne (UNIL) Lausanne Switzerland; ^2^ School of Nursing and Department of Oncology McMaster University Hamilton Ontario Canada; ^3^ Florence Nightingale Faculty of Nursing, Midwifery and Palliative Care King's College London London UK; ^4^ Department of Oncology Lausanne University Hospital (CHUV) Lausanne Switzerland

**Keywords:** early‐phase clinical trial, experience‐based co‐design, oncology

## Abstract

**Background:**

Experience‐Based Co‐Design (EBCD) is a multi‐stage participatory action research process which was developed originally to increase patient involvement in service improvement initiatives. This viewpoint article serves as a reflection on the researchers' experiences, focusing on the application and feasibility of participatory approaches, particularly co‐design, in the specific context of early‐phase clinical trials.

**Methods:**

We reflect on the opportunities and challenges of applying EBCD in a new context of early‐phase clinical trials in oncology where experimental treatments are increasingly perceived as a therapeutic option and, in certain instances, their efficacy may lead to accelerated approval facilitating a swifter integration into standard care.

**Results:**

We propose that the opportunity of applying EBCD in such trials lies in improving the delivery of person‐centered care, care coordination, and support during the transition from experimental to standard care. Three potential challenges when applying EBCD in early‐phase clinical trials are discussed related to: the need for standardization in trial processes; planning EBCD in a context of high uncertainty; and vulnerability of patient populations.

**Conclusion:**

Integrating EBCD into early‐phase oncology trials presents an opportunity to enhance person‐centered care and can lead to simultaneous improvements in care processes and therapeutic development.

**Patient or Public Contribution:**

This article has been developed with the collaboration of a patient partner who serves on the advisory board of our ongoing EBCD study in early clinical trials.

## 1 BACKGROUND

**Figure 1:**
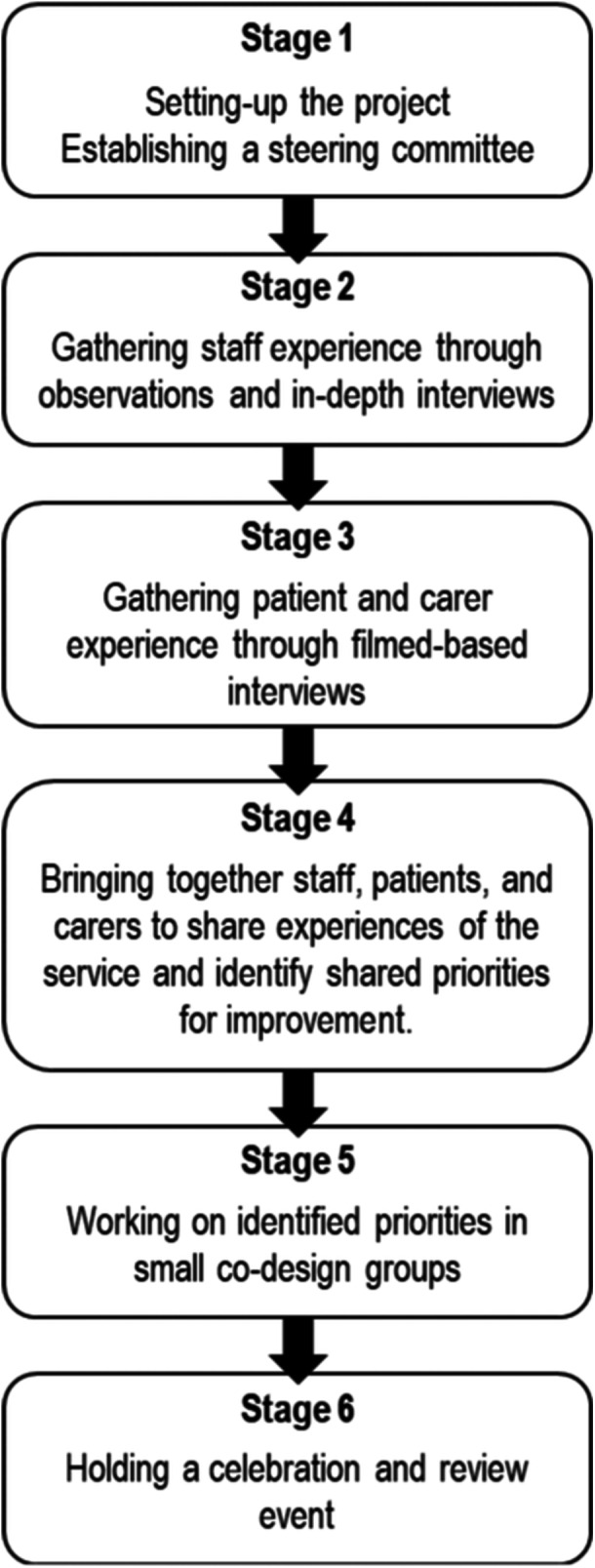
Stages of experience‐based co‐design (adapted from Robert et al.^1^).

Experience‐based co‐design (EBCD) is a form of participatory action research that enables healthcare professionals and patients to identify areas for quality improvement within healthcare settings.^1^ By pinpointing the key moments and situations (or ‘touchpoints') where individuals interact with a service and where their subjective experience is shaped, EBCD provides comprehensive understanding of the challenges and opportunities for enhancing healthcare delivery.^2^ EBCD is a multistage process using qualitative and participatory methods. These methods include observations, individual audio‐recorded and filmed interviews and workshops in which patients and staff work together to co‐design service improvements^3^ (Figure 1). Due to its adaptability, EBCD has been used in various health settings such as primary care,^4^ mental health services^5^ and cancer care.^6^, ^7^ However, to our knowledge, EBCD has never been applied in early‐phase clinical trials in oncology to improve person‐centred care (PCC). Historically, phase 1 clinical trials in oncology have primarily focused on assessing the safety, tolerability and establishing the recommended phase 2 dosage of new treatments, typically involving a limited number of patients. However, with the emergence of immuno‐oncology agents, such as immune checkpoint inhibitors, this traditional approach has recently undergone significant transformation. Clinical trials have incorporated large expansion cohorts within phase 1/2 trials, with the aim of demonstrating not only the safety but also the treatment efficacy of these immunotherapies. This shift has led to conditional accelerated approval for some agents, challenging the traditional phase 1/2/3 drug development process.^8^ The impact of these changes is twofold. First, early‐phase clinical trials are increasingly considered a viable treatment choice for patients facing refractory or relapsed diseases who have exhausted standard therapeutic options.^9^, ^10^ Second, through the accelerated approval process, innovative treatments like immunotherapies swiftly become an integral part of the standard of care for specific cancer types, offering new hope to patients by providing access to innovative therapies with less prolonged delays.^11^

Phase 1 clinical trials are not only characterized by the mobilization of new biological entities or technologies but also by new forms of care adapted to these settings. For instance, the early development of experimental chemotherapies involved ‘total care’, consisting of adjusting diet, psychosocial support and medication.^12^ It has also been argued that supportive or palliative care has to be developed simultaneously to early‐phase clinical trials.^13^ In the contemporary landscape of immuno‐oncology, the boundaries between research and standard care are fading,^14^ which makes attending to the development of new care processes associated with the early clinical uses of an experimental treatment even more relevant. One important consideration is the need to rethink early‐phase surrogate endpoints to ensure that they accurately reflect outcomes that are important to patients.^15^ Additionally, the delivery of supportive care must adapt highly standardized and systematic procedures of trials to a broader group of participants with diverse characteristics and needs. This requires careful consideration of the individual needs and goals of all stakeholders involved to improve the overall quality of care delivery. We are currently implementing the EBCD approach within the context of experimental immunotherapies in early trial phases. Our experiences thus far have provided valuable insights, and we anticipate that sharing our reflections to date could offer assistance and insights to others in similar endeavours (Table 1).

**Table 1:** Overview of our current EBCD study.

Context	Early‐phase clinical trial in immuno‐oncology
Qualitative research	Interviews with patients, informal caregivers and healthcare professionals
	Interviews conducted at different timepoints (inclusion, treatment, follow‐up)
	Observations at different timepoints
Co‐design activities	One workshop to identify key touchpoints and priorities for improvement Mixing up patients, informal caregivers and healthcare professionals Involving only patients who have received the treatment and/or their caregiver One workshop to reach a consensus on a list of recommendations
Patients' involvement	Patients and informal caregivers participating in a co‐design study are not only study participants but also actively engaged in validating qualitative results and developing priorities for service improvement during co‐design workshops
	One patient contributed to the development of participant documentation, including information and informed consent, and ensured the relevance and comprehensibility of the questions in the interview guides
	A second patient representative serves as a member of the study Advisory Board, contributing to the development of the research protocol, engaging in discussions on study progress, participating in result validation analyses, contributing to dissemination/publication efforts (co‐author of this paper) and providing recommendations for implementation

Abbreviation: EBCD, experience‐based co‐design.

Drawing on both existing literature and our ongoing EBCD research experience, we reflect upon: (1) the opportunities of applying EBCD as a method to improve the delivery of PCC in early‐phase clinical trials in oncology; (2) potential challenges to–and solutions for—applying this methodology in such contexts.

## 2 CARE NEEDS AND OPPORTUNITIES OF APPLYING EBCD IN EARLY‐PHASE CLINICAL TRIALS

### 2.1 Person‐Centered Car (PCC)

PCC aims to direct health care around the preferences and needs of patients.^16^ While quality of life and symptom self‐reporting are increasingly measured in early‐phase clinical trials in oncology,^17^ little attention is paid to systematically assess patient needs from a holistic perspective. Given the context of early‐phase clinical trials where patients are exposed to a high physical, mental and spiritual burden,^18^ a more comprehensive understanding of their needs and experiences during these therapies could lead to improvements in the quality of their care and health outcomes. Existing studies have also highlighted the lack of consideration for engaging patients' informal caregivers,^19^ who play a crucial role in supporting patients in early‐phase clinical trials.^20^

EBCD has already demonstrated how it can facilitate the implementation of PCC in oncology by highlighting touchpoints related to information needs about side effects or treatment ending.^6^, ^7^ Furthermore, use of EBCD methods may strengthen the role of informal caregivers in cancer care, such as in the timely reporting of patients' symptoms or seeking professional support when needed.^7^ More widely, EBCD can result in facilitating professionals–patient partnerships by, for instance, developing training and support resources relating to complex care situations.^21^ Therefore, the utilization of EBCD in early‐phase clinical trials can anticipate inherent touchpoints or needs (personal, clinical and organizational) that may pertain not only to the trial itself but also to the therapy being administered. The EBCD approach identifies and prioritizes needs, proposing improvement strategies that will ensure consideration of PCC principles upon treatment approval and standard practice adoption.

### 2.2 Communication related to risks and benefits

Communication between patients and healthcare providers in early‐phase clinical trials continues to pose challenges, encompassing a range of issues, including misinterpretation, confusion and omission of crucial information^22^ as well as the occurrence of therapeutic misconception, wherein patients mistakenly equate research objectives with care goals.^23^ Furthermore, healthcare professionals may find it difficult to explain genuine risks because they want to respect patients' hope in what may be a last curative option.^24^ It has also been documented that patients in early‐phase clinical trials do sometimes not report symptoms for fear of being withdrawn from the experimental protocol.^25^

By valuing users' voices, EBCD may help identify gaps or points to improve regarding communication about the risks, benefits and other sensitive aspects of the clinical trial.^3^ EBCD can improve communication between staff and patients as well as between services,^26^ including when dealing with sensitive information such as adverse reactions or bad news in oncology or palliative care.^4^, ^7^, ^21^ Through bringing patients and staff together as co‐designers, the method has helped to inform the tailoring of information—such as designing information sheets, training or protecting time for communication purposes—to specific organizational contexts.^4^

### 2.3 Lack of care coordination

Existing literature highlights several care coordination issues during early‐phase clinical trials. First, supportive and palliative care are often not well integrated within phase 1/2 clinical trials.^13^ However, it has been argued that ‘simultaneous care’—that is the integration of palliative care within clinical trials—can be instrumental to improving physical, emotional and social well‐being.^13^ Second, a few studies have highlighted a lack of support during the transition between clinical trials and standard care.^27^ This transition is particularly difficult for patients who have been withdrawn from clinical trials, because of health deterioration, violation of a protocol's criteria or a personal decision to withdraw.^28^

EBCD could enhance coordination between supportive or palliative care and clinical trials. Indeed, several studies have shown how EBCD can lead to improvement activities that better integrate different forms of care^5^; for instance, by enhancing the integration of palliative care within an emergency department.^21^ More generally, EBCD seeks to facilitate organizational changes, such as redesigning coordination between teams or departments.^29^

In our study, we engage different professionals beyond the clinical trial team, particularly in the advisory board, including experts in palliative care, psycho‐oncology and social sciences. Involving stakeholders from the outset may enhance their commitment in subsequent stages of implementing the improvement strategies identified.^5^

## 3 POTENTIAL CHALLENGES TO—AND SOLUTIONS FOR—APPLYING EBCD TO CLINICAL TRIALS

### 3.1 Integrating co‐design in a context of high standardization

EBCD seeks to generate change including in complex care settings. However, this could be challenging within the context of clinical trials, which are usually characterized by a high level of standardization. Standardizing practices aim to both organize research procedures and ensure scientific validity through quantification and the reproducibility of research.^30^ The interpretive paradigm of EBCD could become at odds with a ‘traditional, positivist, science paradigm’.^31^ In an EBCD project aiming to improve the experiences of older patients with breast and colorectal cancer, some staff struggled to consider that patients' knowledge could really contribute to design solutions.^4^ Such tensions between research paradigms could represent a barrier to the implementation of EBCD^3^, ^4^; this could be particularly the case in early‐phase clinical trials.

*Solution*

While organizational change may be challenging in the context of the highly standardized practices of clinical trials, it is important to stress that EBCD typically generates ‘liminal’ space for changes.^32^ In other words, EBCD is well suited to both identify and shape new areas within existing services and to enhance communication between stakeholders.^32^ To enhance capacity for change, a solution could lie in the establishment of a steering committee including staff, patients/informal caregivers and institutional representatives to provide support throughout the EBCD project and assure the feasibility and uptake of co‐designed improvements.^7^, ^29^ Professional facilitators can also help support co‐design workshops in complex organizations.^1^

### 3.2 Planning EBCD in a context of high uncertainty

Planning EBCD could be challenging in the context of high uncertainty characterizing early‐phase clinical trials. Indeed, a research protocol can be changed or even interrupted at any time, because a severe adverse reaction has been detected or because a concurrent treatment has demonstrated a higher efficacy.^33^ Uncertainty is also related to recruitment and retention: phase 1 clinical trials are often marked by slow recruitment, failure to reach the inclusion targets or a high rate of patient dropout because of narrow inclusion criteria or overburdening procedures.^34^ Hence, it might be particularly challenging to plan and implement EBCD adequately. For example, it may be challenging to organize joint co‐design workshops when the number of patients who will be recruited and retained in the trial is highly uncertain. Furthermore, staff's time constraints and standardized practices can limit the possibility to conduct each step of the EBCD (although this challenge is not limited solely to the context of clinical trials).

*Solution*

The literature shows that EBCD is a flexible and adaptable method.^5^ One strategy we employed in our study to mitigate the potential impact of low recruitment and retention rates in clinical trials was to utilize a cross‐sectional design for patient inclusion during the study's design phase (Table 3). This implies that patients can be invited to participate in the EBCD study at various stages of the clinical trial, including inclusion, treatment or follow‐up. Employing a purposeful sampling strategy would allow for the inclusion of a predetermined quota of patients at each stage of the clinical trial or a quota of patients responding to treatment or progressing. This not only aims to guarantee an adequate number of participants but also to ensure a diversity of experiences (decision of inclusion, therapeutic failure, benefits, severe adverse reactions, coordination challenges, over‐optimism, noneligibility, etc.), especially during stages 2 and 3 of the EBCD method. Furthermore, involving different patients/informal caregivers at various moments of the clinical trial, and allowing participants to take part in one or several stages of the EBCD process ensures flexibility for both patients, informal caregivers and staff and may enhance the effectiveness and feasibility of the EBCD method (Tables 1 and 2).

**Table 2:** Care needs and opportunities from applying EBCD to early‐phase clinical trials in oncology.

Care needs in early‐phase clinical trials	Opportunities from applying EBCD
person‐centred care	Identify patients' needs
	Enhance the role of informal caregivers
	Envision training activities and support resources in complex care situations
Communication	Improve communication between patients and staff, as well as between services
	Enhance communication related to sensitive information such as the risk of adverse reactions or bad news
	Provide a strategy to tailor information to specific organizational settings
Coordination	Enhance the coordination between services; for instance, between the clinical trial and supportive or palliative care

Abbreviation: EBCD, experience‐based co‐design.

### 3.3 Engaging vulnerable patients and informal caregivers in EBCD

In early‐phase clinical trials in oncology, patients are deemed vulnerable due to the considerable uncertainty surrounding the outcomes of experimental treatments, while it does often represent their last therapeutic option. Patients have a relatively high performance status before entering a phase 1 protocol, while often being confronted with a high symptom burden during the experimental phase.^35^ In addition, many patients may not benefit from treatment,^23^ resulting in poorer physical health and increased psychological distress, especially when hope for an effective final therapeutic option has been dashed. Other studies have also shown the strong psychological impact and moral distress among caregivers of clinical trial participants.^36^, ^37^ Some EBCD studies involving patients with severe conditions or impaired states, such as in palliative care, have documented that recalling their experience can cause mental distress.^21^ Thus, an important issue to consider is the burden of co‐design activities if patients are suffering from severe physical or psychological impairments. Furthermore, involving patients with varying health conditions, outcomes and trial stages during co‐design activities may subject them to divergent realities, causing discomfort and psychological distress.

*Solution*

To overcome specific challenges related to highly vulnerable patients, it is important to minimize the risk of overburdening participants by allowing flexibility and responsiveness to users' needs through meaningful adjustments in EBCD activities (e.g., leverage established community networks, provide a quiet space or emotional support).^38^ Some components can be overlapped or withdrawn (such as the filmed narrative interviews or the observational fieldwork), albeit raising issues in relation to realizing some of the benefits of the approach.^3^ Because the film can be time‐consuming and emotionally challenging to compile, an ‘accelerated’ EBCD approach has been developed and tested based on archives of patient films.^29^ Regarding the ethical challenge of involving vulnerable patients in co‐design activities, available literature emphasizes the need to consider consent as a process that has to be monitored throughout all stages of the research project.^21^ During a clinical trial, patients could encounter physical or psychological challenges that hinder their continuous participation in the various EBCD stages. Seeking clear agreement and willingness to engage before each stage will ensure that ethical standards are followed during the co‐design process. It may also be possible to involve indirectly the most vulnerable patients through patient representatives such as informal caregivers.^21^ As proposed in the previous point, the adoption of a cross‐sectional design and the flexibility to participate in one or multiple EBCD stages could help to alleviate the potential burden associated with participating throughout the entire process while accommodating the diverse needs of patients and research objectives. Whilst patients may be depending on the treatment as their last hope for a therapeutic option, this may make it particularly problematic to engage in co‐design activities (steps 4, 5 and 6). Hence, attention should be directed towards avoiding the integration of (a) patients who have benefitted from the treatment and/or their informal caregivers with (b) other patients and/or their informal caregivers in a situation of treatment failure, dropout or withdrawal or who could not receive the therapy (e.g., disease progression, health deterioration, manufacturing‐related issues). In this regard, independent co‐design workshops or alternative strategies, such as individual sessions with each patient to identify priorities and strategies, could be considered. As part of the latter approach, individual validation and rating systems for the overall results could be implemented, even remotely. Table 3 provides a summary on the challenges and solutions discussed in this section.

**Table 3:** Potential challenges to—and solutions for—applying EBCD to early‐phase clinical trials in oncology.

Potential challenges	Potential solutions	EBCD stages
Integrating co‐design in a context of high standardization and positivist science paradigm	Establish a steering committee to engage main stakeholders throughout the study to help ensure the feasibility and uptake of co‐designed improvements	Transversal
	Use trained facilitators for co‐design activities	4, 5, 6
Planning EBCD in a context of high uncertainty	Purposive sampling based on cross‐sectional design to guarantee an adequate number of participants but also to ensure a diversity of experiences	2, 3
	Flexibility to participate in one or several EBCD stages	Transversal
Involving vulnerable patients and/or their informal caregivers in co‐design activities	Constantly monitor consent processes at each EBCD stage	Transversal
	Adapt the method by, for example, removing a component or overlapping steps (e.g., ‘accelerated’ designs relying on film archives)	Transversal
	Indirectly involve in co‐design the most vulnerable patients through patients' representatives; restricted use of excerpts of interviews and films	4, 5, 6
	Purposive sampling based on cross‐sectional design to assure diversity of experiences and avoid risk of overburdening through a longitudinal design	2, 3
	Allow flexibility and responsiveness to participant needs	Transversal
	Run parallel co‐design processes with distinct groups to focus on their specific experiences and bring the findings together when implementing and testing solutions	4, 5, 6
	Think about alternative ways of including patients in instances of treatment failure, dropouts, withdrawals or inability to receive therapy; for instance, through individual validation and rating systems for the overall results, even remotely	4, 5, 6

Abbreviation: EBCD, experience‐based co‐design.

Lastly, while it is true that participants in this context are particularly vulnerable, we have to emphasize that the desire to help future patients is a strong motivation to participate in early‐phase clinical trials,^23^ and therefore in EBCD as a means to improve care delivery and services.

## 4 DISCUSSION

In the context of early‐phase clinical trials in oncology, it is increasingly important to anticipate care needs before an experimental treatment should be used in routine practice. Due to its transformative nature in complex health settings,^3^ EBCD represents a way to develop more PCC simultaneously to the development of an experimental therapy. However, the experimental settings of clinical trials could pose specific challenges for using the EBCD approach. High standardization settings are likely to increase the challenges of engaging all stakeholders and their undertaking improvement activities. In a context of uncertainty about the duration of a clinical trial, planning each step of EBCD could be particularly challenging. We propose that as a flexible method used widely with vulnerable patients, adaptations to the standard EBCD approach can overcome these challenges. In this manuscript, we suggest potential solutions and alternative strategies to overcome them. While the early stages of the EBCD approach ensure the diversity of individual experiences (gathered by qualitative methods), special attention needs to be paid to the challenge of upbringing together patients in the co‐design stages at very different stages of the trajectory of care or with different outcomes. Although the possibility of patients dropping out and different patients participating in various EBCD stages might be seen as a challenge, it can actually enrich the process by bringing diverse perspectives and experiences. This diversity allows for a more comprehensive exploration of the issues at hand and promotes inclusivity in the development of solutions. Variation in participant involvement can ultimately enhance the effectiveness and relevance of the iterative co‐design stages of the EBCD approach.

In contrast to traditional qualitative research methods, EBCD offers distinct advantages by not only capturing diverse individual care experiences but also facilitating consensus‐building and co‐created targeted strategies and solutions for improvement. Although alternative methods or designs—such as participatory action research, focus groups or collaborative brainstorming sessions—can ensure the involvement of all stakeholders, EBCD provides a structured framework to guide and implement solutions, ensuring a systematic process from exploring individual experiences to driving meaningful change.

## AUTHOR CONTRIBUTIONS

**Nils Graber**: Investigation; writing—original draft; methodology; writing—review & editing. **Nina Canova**: Investigation; writing—review and editing; methodology. **Denise Bryant‐Lukosius**: Conceptualization; validation; writing—review and editing. **Glenn Robert**: Conceptualization; methodology; writing—review and editing. **Blanca Navarro‐Rodrigo**: Conceptualization; validation. **Lionel Trueb**: Validation. **George Coukos**: Conceptualization; validation. **Manuela Eicher**: Conceptualization; writing—review and editing; funding acquisition. **Tourane Corbière**: Validation; writing—review and editing; conceptualization. **Sara Colomer‐Lahiguera**: Conceptualization; funding acquisition; investigation; writing—original draft; methodology; writing—review & editing.

## CONFLICT OF INTEREST STATEMENT

The authors declare no conflict of interest.

## Data Availability

Data sharing is not applicable to this article as no data sets were generated or analysed during the current study.
